# *Leclercia adecarboxylata* in Peritoneal Dialysis Patients: A Systematic Review

**DOI:** 10.3390/pediatric15020025

**Published:** 2023-04-25

**Authors:** John Dotis, Antonia Kondou, Vasiliki Karava, Georgia Sotiriou, Athina Papadopoulou, Charalampos Zarras, Chrysi Michailidou, Eleni Vagdatli, Nikoleta Printza

**Affiliations:** 1 First Department of Pediatrics, Medical Faculty, School of Health Sciences, Aristotle University of Thessaloniki, Hippokration General Hospital, 54642 Thessaloniki, Greece; 2 Department of Microbiology, Medical Faculty, School of Health Sciences, Aristotle University of Thessaloniki, Hippokration General Hospital, 54642 Thessaloniki, Greece

**Keywords:** *Leclercia adecarboxylata*, peritonitis, review

## Abstract

**Background:** *Leclercia adecarboxylata* is a Gram-negative bacillus that can rarely cause infections in humans. We recently treated a case of peritonitis due to *L. adecarboxylata* in a peritoneal dialysis (PD) pediatric patient, and we systematically reviewed all the relevant reported cases in the literature. **Methods:** We searched the PubMed and Scopus databases, and we reviewed 13 such cases (2 children, 11 adults) that were reported, including our patient. **Results:** The mean (±SE) age was 53.2 ± 22.5 years, with a male-to-female ratio of approximately 1:1.6. Their mean vintage period on PD prior to *L. adecarboxylata* peritonitis was 37.5 ± 25.3 months. The VITEK card was the identification diagnostic tool in most cases (63%). The antimicrobial agent that was most frequently used was ceftazidime, which was implemented in 50% of cases as initial therapy, either as a monotherapy or combination therapy; in only two patients (15.3%) was the Tenkhoff catheter removed. The median duration of treatment was 18 days (range of 10–21 days), and all 13 patients that were reviewed were healed. **Conclusions:** Physicians should be aware that *L. adecarboxylata* is noted to rarely cause peritonitis in PD patients; however, this pathogen seems to be sensitive to most antimicrobial agents and can result in a favorable outcome with the selection of appropriate treatment.

## 1. Introduction

*Leclercia adecarboxylata* is a Gram-negative bacterium belonging to the family Enterobacteriaceae [1]. *L. adecarboxylata* is a member of the normal gut flora in animals and has also been isolated from the human gut [2]. It can rarely cause infections in humans, especially in immunocompromised individuals [3]. The majority of clinical isolates of *L. adecarboxylata* are susceptible to commonly used antibiotics; however, resistant strains producing extended-spectrum beta-lactamases (ESBLs) have been recently reported [4].

Peritonitis is a very serious complication, often responsible for catheter loss and switching the dialysis modality in patients undergoing peritoneal dialysis (PD) due to end-stage kidney disease [5]. There has been increased recognition of peritonitis caused by rare organisms mainly due to better and updated identification methods. However, peritonitis cases due to *L. adecarboxylata* in PD patients are presented extremely rarely in the worldwide literature.

We recently encountered a case of peritonitis due to *L. adecarboxylata* in a young PD patient with a favorable outcome, and we have since systematically reviewed the international literature for other such cases with the aim of enriching our knowledge about this rare and noteworthy pathogen.

## 2. Case Report

A 14.5-year-old immunocompetent male started treatment with PD because of corticosteroid-resistant nephrotic syndrome, specifically focal segmental glomerulonephritis, 4 years ago. Although he had two previous peritonitis episodes at 11.5 years old and 13.5 years old, both due to *Staphylococcus* spp., he followed a nocturnal intermittent PD (NIPD) program without any ultrafiltration problem during these years. Before admission, he developed signs and symptoms consistent with peritonitis, such as cloudy peritoneal effluent, low-grade fever (38.4 °C) for 8 h, abdominal pain, and vomiting. Microbiologic investigation of the peritoneal effluent revealed a white cell count of 3700/μL, with a polymorphonuclear (PMN) cell ratio of 82%.

No tenderness or cutaneous lesions were elicited along the tunnel of the PD catheter. The patient was treated empirically with intraperitoneal ceftazidime (125 mg/L) plus vancomycin (30 mg/L) while no organisms were revealed in the Gram stain. On day 4, the PD effluent culture from the time of his admission was reported to be growing *L. adecarboxylata* by a VITEK-II GN card (bioMérieux, Marcy l’Etoile, France) and by conventional tests. In vitro susceptibility testing was performed by a Kirby–Bauer disc diffusion test on Mueller–Hinton agar according to CLSI guidelines [6]. The cultured pathogen was found to be sensitive to all antibiotics tested. Specifically, the agent’s susceptibility test showed susceptible to amikacin (MIC ≤ 2 mg/L), amoxicillin/clavulanic acid (MIC ≤ 2 mg/L), ampicillin (MIC ≤ 2 mg/L), cefotaxime (MIC ≤ 1 mg/L), ceftazidime (MIC ≤ 1 mg/L), ciprofloxacin (MIC ≤ 0.25 mg/L), ertapenem (MIC ≤ 0.5 mg/L) and piperacillin/tazobactam (MIC ≤ 4 mg/L). Vancomycin was discontinued, and treatment monotherapy with intraperitoneal ceftazidime (125 mg/L) was given for a total of 21 days. The patient responded favorably without catheter replacement. During the episode of peritonitis, the NIPD program was temporarily changed to continuous ambulatory PD (CAPD) for two days and then switched again to NIPD. When re-assessed one week, one month and three months after the end of treatment, he was well, without any signs or symptoms of peritonitis or ultrafiltration problems.

## 3. Subjects and Methods

### 3.1. General Information and Literature Search Strategy

This review conforms to the Preferred Reporting Items for Systematic Reviews and Meta-Analyses (PRISMA) statement [7,8] (PROSPERO 2022 CRD42022307978; available from https://www.crd.york.ac.uk/prospero/display_record.php?ID=CRD42022307978; accessed on 13 September 2022).

### 3.2. Eligibility Criteria

This review includes case reports of PD patients that followed definitions of *L. adecarboxylata* peritonitis. The diagnosis of peritonitis was based on criteria including clinical manifestations such as abdominal pain, nausea and fever combined with a cloudy peritoneal effluent count of 100 WBC/µL or greater, consisting of at least 50% PMN cells. In addition, peritonitis was defined as a case presenting with a positive culture for *L. adecarboxylata* from peritoneal fluid [5]. The identification of *L. adecarboxylata* was made either by conventional tests, by an automated system, or by whole-genome sequencing.

### 3.3. Information Sources and Search Strategy

We searched the literature for cases of human infection with *L. adecarboxylata*. Articles were obtained from two databases: PubMed (US National Library of Medicine National Institutes of Health, www.ncbi.nlm.nih.gov/pubmed, accessed on 1 December 2022) and Google Scholar (Google, www.scholar.google.com, accessed on 1 December 2022). Databases were searched using the keywords “*Leclercia adecarboxylata*” and “peritonitis” and “peritoneal dialysis” in any available language. For the translation of text, we used the Google online translation tool (Google, https://translate.google.gr, accessed on 3 January 2023).

### 3.4. Study Selection

All potentially relevant articles published through 31 October 2022 were screened in two stages for eligibility by selected authors based on their previous experience in such reviews. In the first stage of assessment, the titles and abstracts of potentially relevant articles were screened independently by three authors (JD, AK, GS). The reference list for each article was examined to verify that all published cases had been collected. For those abstracts that met the inclusion criteria, the full text was retrieved and independently reviewed by two authors in the second stage of assessment (VK, NP). Disagreements and technical uncertainties were discussed and resolved by all authors (JD, AK, VK, GS, AP, CZ, CM, EV, and NP).

### 3.5. Data Extraction

The primary citations obtained during the database survey were recorded in a text file according to their topics and abstracts. None of the case reports found were excluded from enrolment in the analysis due to the quality or inadequacy of data reported, although in some reports, all data were not available. Variables included in the database were the year of publication, origin, demographic information (gender and age), underlying disease, previous peritonitis episodes, symptoms, temperature levels and laboratory findings during admission, predisposing factors, diagnosis method, and *L. adecarboxylata* sensitivity patterns. In addition, data on treatment choice, duration and route of treatment, catheter removal procedure, and outcome were recorded.

### 3.6. Ethics Statement

Due to the retrospective, literature review nature of the study, the Ethics Committee of Hippokration Hospital of Thessaloniki, Greece, determined that patients’ consent was not required. In addition, we have ensured that our patient’s data are kept confidential and in compliance with the Declaration of Helsinki.

### 3.7. Statistical Analysis

All articles found were systematically reviewed, and a master database was constructed. Microsoft Excel software (XP Professional) version 5.2.3790.1830 (Redmond, WA, USA) was used to develop this database of categorical and continuous variables. The statistical program Graphpad Instat 3.10 (Graphpad Inc., San Diego, CA, USA) was used. A two-sided *p*-value of <0.05 indicated statistical significance.

## 4. Results

**Figure 1:**
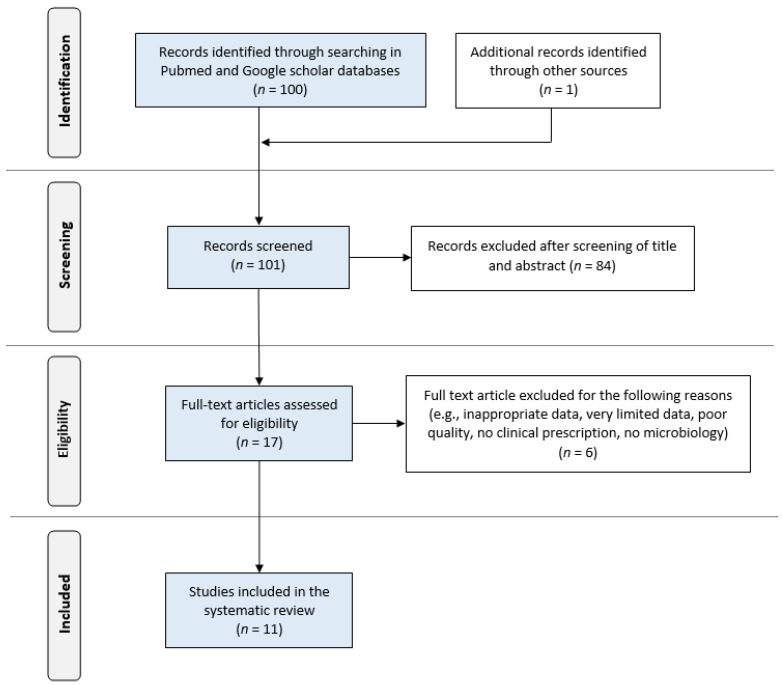
PRISMA flow diagram of literature search, eligibility and inclusion process.

The systematic search, as illustrated in Figure 1, resulted in an initial number of 101 potentially relevant articles. After the screening, the remaining 12 publications fulfilled the eligibility criteria and were included in this review. Of these, 11 were case reports, and 1 was a case series [3,9,10,11,12,13,14,15,16,17,18,19]. Twelve cases of peritonitis due to *L. adecarboxylata* in PD patients have been reported so far in the literature in all languages, to which we contributed a new case, bringing the total to 13. The first case was described in 1998, while out of the 13 cases, 3 (23%) were reported from the USA and Korea, respectively, and 1 case (8%) each was reported from France, Greece, India, Portugal, Spain, Taiwan, and Turkey.

Case reports of these patients, including their demographic and clinical characteristics, are enumerated in Table 1. Their mean (±SE) age was 53.2 ± 22.5 years, with a male-to-female ratio of approximately 1:1.6. These comprised 11 adults (>18 years, mean age ± SE, 61.1 ± 12.6 years) and 2 children (<18 years, median age 9.75, range 5–14.5 years). The mean vintage period on PD prior to *L. adecarboxylata* peritonitis was 37.5 ± 25.3 months, while the most common underlying disease was diabetic nephropathy in 71.4% of 7 patients with available data.

The PD effluent was cloudy in 8 cases with all data presented. The mean peritoneal white blood cell count during admission was 8.859/mm^3^ (range 1250–48,000/mm^3^), with a mean value of 85% PMN cells. Abdominal pain was the predominant symptom in all cases, followed by vomiting, nausea and fever, in their order of appearance. The mean temperature was 38.4 °C in 6 patients with available data. The identification diagnostic tools for each *L. adecarboxylata* case are presented in Table 2. In most cases (63%), a VITEK card established the diagnosis, while the initial Gram stain was suggestive of *L. adecarboxylata* in only two cases.

The management of PD patients with peritonitis due to *L. adecarboxylata* is summarized in Table 3. All patients received antimicrobial treatment; however, in one patient, data were not presented. Of note was the fact that in only two patients (15.3%) was a catheter removal procedure performed. In most cases, the administration of antimicrobial agents intraperitoneally was followed by intravenous administration. The antimicrobial agent that was most frequently used as initial therapy, either empirically or etiologically, was ceftazidime. Specifically, in 6/12 (50%) cases, ceftazidime was the initial therapy, either as a monotherapy or combination therapy. In 5/6 (83%) of cases, initial therapy with ceftazidime was continued with the same agent until the end of treatment. Other used agents were amikacin, gentamicin, cefpiramide, cefazolin, tobramycin, ciprofloxacin and imipenem. Vancomycin was used as an initial, empirical treatment in a few cases; however, after the identification of *L. adecarboxylata*, this glycopeptide antibacterial agent was discontinued. The median duration of treatment was 18 days, with a range between 10 and 21 days. Remarkably, outcomes were favorable in all 13 patients that were reviewed in this study.

## 5. Discussion

This comprehensive review of 13 PD patients highlights the notion that *L. adecarboxylata* is an emerging pathogen that can cause peritoneum infections, is susceptible to most antibiotics, and is relatively non-life-threatening. Most certainly, *L. adecarboxylata* constitutes a ubiquitous, motile, facultative, anaerobic, Gram-negative bacillus of the *Enterobacteriaceae* family. Isolation of this organism comes from multiple sources in nature, including water, and it can also be a part of normal flora in animals and humans [2]. Although often acquired via a wound, which may be combined or not with an aquatic environment, in our patient, the source of infection was unidentified. *L. adecarboxylata* can rarely cause infections, including endocarditis, catheter-related bacteremia, skin and soft tissue infections, bacteremia, pneumonia, meningitis, urinary tract infections, spontaneous bacterial peritonitis and, of course, peritonitis in PD patients [3,20].

Of note is the fact that prompt diagnosis of *L. adecarboxylata* can be delayed due to similar biochemical properties as *Eschericia coli* strains. Particularly, *L. adecarboxylata,* unlike *Eschericia* strains, are occasionally positive for urease hydrolysis, grow in the presence of potassium cyanide, and are positive for malonate utilization and yellow pigment production and, by contrast, are negative for lysine and ornithine decarboxylase tests [21]. It is important to remark that if the conventional diagnostic methods are exceeded, the introduction of automated identification systems decreases the risk of misidentification between *L. adecarboxylata* and *E. coli* strains. Specifically, automated systems such as VITEK, WIDE API and BD Phoenix, with the addition of the latest matrix-assisted laser desorption ionization time-of-flight (MALDI-TOF) technology, play a crucial role in the identification of uncommon bacteria and in establishing their real incidence [22].

Current empiric regimens for peritonitis caused by Gram-negative pathogens in PD patients most commonly recommend the IP use of a third- or fourth-generation cephalosporin, such as ceftazidime or cefepime, respectively. In addition, quinolones, such as oral ciprofloxacin, can be an alternative choice of treatment [5]. It is remarkable that in several reports, *L. adecarboxylata* isolates were revealed to be pansensitive or sensitive to most antimicrobial agents tested, including beta-lactam antibiotics, aminoglycosides, quinolones, and tetracyclines [20]. Nevertheless, resistance was also found in other studies, and even more rarely, ESBL strains have been found that constitute a therapeutic problem for clinicians [4,23]. *L. adecarboxylata* multidrug-resistant strains can become life-threatening human bacterial pathogens by including blaTEM-1 and blaCTX-M group 1 and intl1 genes (dfrA12-orfF-aadA2) as genetic determinants for resistance [24].

A suggestion to remove the Tenckhoff catheter in peritonitis caused by *L. adecarboxylata* cannot yet be established, but some evidence suggests removal was necessary in a few cases [16,18]. However, the ability of *L. adecarboxylata* to produce a biofilm is unknown, although the association of *L. adecarboxylata* with catheter-related septicemia, particularly tunneled central venous catheters, is reported to be increased [20]. In addition, considering the fact that the Tenckhoff catheter was removed in only two of the reported cases, in which one had relapsing peritonitis episodes, any recommendation for removal is currently problematic and unconfirmed.

A limitation of this review was the bias of analysis of published cases, as there is more incentive to publish cases that were successfully treated, and this probably explains why all of our reviewed cases had a favorable outcome. Despite this limitation, the focus of this review was mainly on the clinical, diagnostic, and microbiological features and the treatment of *L. adecarboxylata* peritonitis. Unfortunately, this review does not reflect the true incidence or prevalence of peritonitis due to *L. adecarboxylata* and is insufficient to draw definitive conclusions or make generalizations about the management of this pathogen. In addition, since this is a case series study, there is no comparison group to evaluate the effectiveness of the treatment strategies used. Therefore, it is difficult to determine if the outcomes were solely due to the treatment provided or other factors. Furthermore, the study did not perform any statistical analysis to evaluate the significance of the findings. Therefore, it is unclear if the treatment strategies used were effective or if the outcomes were simply due to chance. However, based on the rarity of the infection, it is important to collect information from individual cases so that conclusions about predisposing factors, microbiology aspects, and the preferable treatment options can be safely drawn.

## 6. Conclusions

In conclusion, the prompt diagnosis of *L. adecarboxylata* with the usage of appropriate microbiologic identification diagnostic tools might increase the prevalence of this rare microorganism as a cause of peritonitis in PD patients. In addition, it has been speculated that the real number of *L. adecarboxylata* infections could be under-reported due to the high degree of phenotypic overlap between *L. adecarboxylata* and *E. coli* strains. This pathogen seems to be pansensitive or sensitive to most antimicrobial agents; however, healthcare providers should have all these considerations in mind when treating patients with infections caused by this uncommon microorganism in order to achieve a favorable outcome.

## Figures and Tables

**Table 1:** Demographic and clinical characteristics of peritonitis due to *Leclercia adecarboxylata* in peritoneal dialysis patients.

	Reference, Publication Year, Language	Country	Age (y)	Gender	Vintage on PD (Months), (Etiology)	PD Method	Previous Peritonitis
1	[9], 1998, (Korean)	Korea	60	M	NA	CAPD	NA
2	[10] *, 2000, (English)	USA	5	M	9, (NA)	CDPD	2 m previous (coagulase-negative *Staphylococcus*)
3	[11] *, 2001, (Spanish)	Spain	74	M	NA	NA	NA
4	[12] *, 2009, (Korean)	Korea	56	M	24, (diabetic ESRD)	APD	No
5	[13], 2009, (Korean)	Korea	60	F	7, (diabetic ESRD)	NA	No
6	[14], 2013, (English)	Portugal	77	F	51, (NA)	CAPD	1
7	[15] *, 2014, (English)	Taiwan	48	F	24, (diabetic ESRD)	APD	NA
8	[16] *, 2016, (English)	India	38	F	44, (NA)	CAPD	NA
9	[17] *, 2017, (English)	Turkey	72	F	60, (chronic glomerulonephritis)	CAPD	NA
10	[18] *, 2017, (English)	USA	68	F	84, (diabetic ESRD)	CCPD	Multiple prior episodes of PD peritonitis
11	[19] *, 2019 (English)	USA	48	F	24, (diabetic ESRD)	APD	No
12	[3] *, 2021, (English)	France	71	F	NA	NA	NA
13	2022, (English)	Greece	14.5	M	48, (focal segmental glomerulonephritis)	NIPD	1st 11.5 years old, 2nd 13.5 years old (both due to *Staphylococcus* spp.)

PD, peritoneal dialysis; M, male; F, female; ESRD, end-stage renal disease; CAPD, continuous ambulatory peritoneal dialysis; CDPD, continuous daily peritoneal dialysis; CCPD, continuous cycling peritoneal dialysis; APD, automated peritoneal dialysis; NIPD, nocturnal intermittent peritoneal dialysis; NA, not available. * indexed medical journals.

**Table 2:** Diagnostic tools for the detection of *Leclercia adecarboxylata*.

Case	Identification Diagnostic Tool
1	NA
2	VITEK GN card (BioMerieux, Hazelwood, MO, USA)
3	WIDE API 32 GN (BioMerieux, Marcy l’Etoile, France)
4	VITEK-II GN card (BioMerieux, Marcy l’Etoile, France)
5	VITEK GN card (BioMerieux, Hazelwood, MO, USA)
6	NA
7	NA
8	BD Phoenix system (BD Diagnostic Systems, Sparks, MD, USA)
9	NA
10	NA
11	VITEK MS—Matrix Assisted Laser Desorption Ionization Time-of-Flight (BioMerieux, Marcy l’Etoile, France)
12	Matrix-Assisted Laser Desorption Ionization Time-of-Flight (Biotyper-Microflex, Bruker Daltonics, Bremen, Germany)
13	VITEK-II GN card (bioMérieux, Marcy l’Etoile, France)

NA, not available; GN, Gram-negative.

**Table 3:** Management of peritonitis due to *Leclercia adecarboxylata* in peritoneal dialysis patients.

Case	Treatment	Catheter Removal
1	cephalothin + amikacin/ip	No
2	ceftazidime + gentamicin/iv + ip for 10 days	No
3	NA	No
4	ceftazidime/ip for14 days	No
5	cefpiramide/ip for 12 days	No
6	ceftazidime/ip for 15 days	No
7	cefazolin/ip	No
8	tobramycin + cefazolin/ip => amikacin/ip	Yes
9	cefuroxime/ip, ciprofloxacin/pos => imipenem/iv for 21 days	No
10	vancomycin + ceftazidime/ip => ceftazidime/ip for 21 days	Yes
11	vancomycin + ceftazidime/ip => cefazolin/ip for 21 days	No
12	amoxicillin/iv	No
13	vancomycin + ceftazidime/ip => ceftazidime/ip for 21 days	No

iv, intravenous; ip, intraperitoneal; pos, per os; NA, not available.

## Data Availability

Data available on request due to restrictions, e.g., privacy or ethical.

## References

[1] Tamura K., Sakazaki R., Kosako Y., Yoshizaki E. (1986). *Leclercia adecarboxylata* Gen. Nov., Comb. Nov., formerly known as *Escherichia adecarboxylata*. Curr. Microbiol..

[2] Hess B., Burchett A., Huntington M.K. (2008). *Leclercia adecarboxylata* in an immunocompetent patient. J. Med. Microbiol..

[3] Zayet S., Lang S., Garnier P., Pierron A., Plantin J., Toko L., Royer P.Y., Villemain M., Klopfenstein T., Gendrin V. (2021). *Leclercia adecarboxylata* as Emerging Pathogen in Human Infections: Clinical Features and Antimicrobial Susceptibility Testing. Pathogens..

[4] Alosaimi R.S., Muhmmed Kaaki M. (2020). Catheter-Related ESBL-Producing *Leclercia adecarboxylata* septicemia in hemodialysis patient: An emerging pathogen?. Case Rep. Infect. Dis..

[5] Li P.K., Chow K.M., Cho Y., Fan S., Figueiredo A.E., Harris T., Kanjanabuch T., Kim Y.L., Madero M., Malyszko J. (2022). ISPD peritonitis guideline recommendations: 2022 update on prevention and treatment. Perit. Dial. Int..

[6] Clinical and Laboratory Standards Institute (2018). Performance Standards for Antimicrobial Susceptibility Testing.

[7] Moher D., Liberati A., Tetzlaff J., Altman D.G. (2009). Preferred reporting items for systematic reviews and meta-analyses: The PRISMA statement. J. Clin. Epidemiol..

[8] Stewart L.A., Clarke M., Rovers M., Riley R.D., Simmonds M., Stewart G., Tierney J.F., PRISMA-IPD Development Group (2015). Preferred reporting items for systematic review and meta-analyses of individual participant data: The PRISMA-IPD statement. JAMA.

[9] Hwang H.Y., Jeong S.H., Rim H., Rim H., Kim M.H., Jeong T.J., Choi B.G. (1998). A case of *Leclercia adecarboxylata* isolated from dialysate in a patient with continuous ambulatory peritoneal dialysis. Korean J. Clin. Microbiol..

[10] Fattal O., Deville J.G. (2000). *Leclercia adecarboxylata* peritonitis in a child receiving chronic peritoneal dialysis. Pediatr. Nephrol..

[11] Rodríguez J.A., Sánchez F.J., Gutiérrez N., García J.E., García-Rodríguez J.A. (2001). Bacterial peritonitis due to *Leclercia adecarboxylata* in a patient undergoing peritoneal dialysis. Enferm. Infecc. Microbiol. Clin..

[12] Lee H.N., Park J.W., Kim H.S., Park S.H., Chang J.H., Chung W.K., Lee H.H., Seo Y.H., Kim S. (2009). Peritonitis due to *Leclercia adecarboxylata* in a patient receiving automated peritoneal dialysis. Kidney Res. Clin. Pract..

[13] Lee W., Yi D.Y., Jung B., Huh J.Y., Kang M.S., Hong S.G., Hong S.K. (2009). Two cases of independent infection by *Leclercia adecarboxylata*. Infect. Chemother..

[14] Farinha A., Vaz A., Assunção J., Vinhas J. (2013). Unusual bacteria causing peritonitis in peritoneal dialysis—A single centre experience. Port. J. Nephrol. Hypert..

[15] Chao C.T., Hung P.H., Huang J.W., Tsai H.B. (2014). Cycler cassette rupture with *Leclercia adecarboxylata* peritoneal dialysis peritonitis. Perit. Dial. Int..

[16] Ghosh R., Misra R., Prasad K.N., Prasad N. (2016). Peritonitis by *Leclercia adecarboxylata* in a patient with continuous ambulatory peritoneal dialysis: The first case report from India. Int. J. Res. Med. Sci..

[17] Atas D.B., Velioglu A., Asicioglu E., Arikan H., Tuglular S., Ozener C. (2017). Polymicrobial peritonitis with *Leclercia adecarboxylata* in a peritoneal dialysis patient. Saudi J. Kidney Dis. Transpl..

[18] Hobby G., Mandavilli K., Singh M. (2017). A Case report of *Leclercia adecarboxylata* peritonitis in a peritoneal dialysis patient with review of the literature. Int. J. Nephrol. Kidney Fail..

[19] Adapa S., Konala V.M., Nawaz F., Javed T., Dhingra H., Gutierrez I.A., Ramirez M.L. (2019). Peritonitis from *Leclercia adecarboxylata*: An emerging pathogen. Clin. Case Rep..

[20] Spiegelhauer M.R., Andersen P.F., Frandsen T.H., Nordestgaard R.L.M., Andersen L.P. (2019). *Leclercia adecarboxylata*: A case report and literature review of 74 cases demonstrating its pathogenicity in immunocompromised patients. Infect. Dis..

[21] Anuradha M. (2014). *Leclercia adecarboxylata* isolation: Case reports and review. J. Clin. Diagn. Res..

[22] Feucherolles M., Cauchie H.M., Penny C. (2019). MALDI-TOF Mass spectrometry and specific biomarkers: Potential new key for swift identification of antimicrobial resistance in foodborne pathogens. Microorganisms.

[23] Mazzariol A., Zuliani J., Fontana R., Cornaglia G. (2003). Isolation from blood culture of a *Leclercia adecarboxylata* strain producing an SHV-12 extended-spectrum beta-lactamase. J. Clin. Microbiol..

[24] Shin G.W., You M.J., Lee H.S., Lee C.S. (2012). Catheter-related bacteremia caused by multidrug-resistant *Leclercia adecarboxylata* in a patient with breast cancer. J. Clin. Microbiol..

